# Legal Test of Claims for Medical Services at Inquest, and Expert Testimony

**Published:** 1887-11

**Authors:** J. E. Mayfield

**Affiliations:** Nacogdoches, Texas


### LEGAL TEST OF CLAIMS FOR MEDICAL SERVICES AT INQUESTS, AND EXPERT TESTIMONY.

*Reported for Daniel's Texas Medical Journal, by J. E. Mayfield, M. D., Nacogdoches, Texas.*

This is strictly a test case, by Dr. Jno. B. Fears, a regular and highly respectable general practitioner, living and practicing near the village of Garrison, in the northern part of Nacogdoches county, Texas. Dr. Fears was twice compelled, by officers, to leave home and business, ride seven miles, spend one entire day, extending far into the night, make post mortem examinations, and give testimony in the trials. The trials of the murderers will require his attendance at the District Court, twenty miles from his home and business, "from day to day, and from term to term," giving expert testimony.

He presented an account for these services, twenty dollars in each case, but was allowed by the commissioners' court only five dollars each. He appealed to the justice's court, and got judgment for same amount, the costs lost. He then appealed to the District Court, with same result;—and now he carries it to the Superior Court. His object is more to test the merits of such cases than to recover fees. The case has already cost him far more than the amount of his claim, besides much valuable time and trouble. He is represented by able counsel, and the judge who tried the case is of the very highest ability. The following explains itself:

(Copied from the Original.)

J. B. FEARS vs. NACOGDOCHES COUNTY. } *In the District Court, Nacogdoches County, Texas, October Term, 1887.*

In this case the Judge files the following as his findings of facts, from the evidence adduced on the trial, viz:

1. That at the time of the alleged services, plaintiff was a practicing physician and surgeon, and that, as such, he was summoned by a justice of the peace, as a witness to testify as an expert at the inquests upon the dead bodies of William Erwin and Sailor Scoggins; and that, as incidental to his giving testimony, and to enable him the better to do so, he was required to cut a bullet from the leg and to probe a wound in the abdomen of Erwin, and to probe a wound in the face of Scoggins; that, while these operations upon dead bodies, perhaps, required no surgical skill, yet, that their performance by a surgeon, and testimony in relation to the wounds by him, was more satisfactory to the officer and jury than from an unskilled person.

2. That he was engaged but a few minutes less than one hour, in the performance of each operation, but that he lost as much as one day's time in attending each inquest.

3. That the value of his services, estimated upon the basis of legal witness fees (in cases where allowed), would be $1.50 in each case, and upon the basis of a juror, $2.00 in each case, and upon the basis of reasonable charges made by surgeons in private cases, $20.00 in each case.

4. That the claim sued for was presented to the commissioners' court before this suit was begun, and allowed by said court for $5.00 in each case, being $10.00 in the aggregate, and rejected, as to the balance, and that no part of said claim has been paid.

### FINDINGS OF LAW.

Upon the foregoing facts, the Judge files the following as his findings of the law, viz:

That he is inclined to the opinion, and believes, perhaps, the correct law of the case to be that plaintiff attended the inquests and did the acts which he calls services, and for which he claims compensation, in the capacity of an ordinary expert witness, and that he is entitled to no other compensation than legal witness fees, if it were a case in which such fees were allowed; if not such case, then no compensation; but, resolving the doubt as to the correctness of this view of the law in favor of plaintiff, the court finds, as its conclusion of law, that the services of plaintiff were of a quasi public character, similar in nature to those required of witnesses, jurors, etc., for the public good, and that the compensation to which plaintiff is entitled for the same, should be estimated upon the basis of such analogies, and not upon the basis of what would be a reasonable charge for same, as between individuals, and that, this basis held by the court to be correct, the plaintiff is entitled to $5.00 for his services at each inquest, making together the sum of $10.00, which amount the plaintiff is entitled to recover, and judgment is rendered for plaintiff for said sum, and costs of suit adjudged against plaintiff, because judgment here recovered is for no larger amount than recovered by plaintiff in commissioners' court.

JAMES I. PERKINS, Judge Presiding.

